# Cost-effectiveness of adding oseltamivir to primary care for influenza-like-illness: economic evaluation alongside the randomised controlled ALIC^4^E trial in 15 European countries

**DOI:** 10.1007/s10198-022-01521-2

**Published:** 2022-09-22

**Authors:** Xiao Li, Joke Bilcke, Alike W. van der Velden, Robin Bruyndonckx, Samuel Coenen, Emily Bongard, Muirrean de Paor, Slawomir Chlabicz, Maciek Godycki-Cwirko, Nick Francis, Rune Aabenhus, Heiner C. Bucher, Annelies Colliers, An De Sutter, Ana Garcia-Sangenis, Dominik Glinz, Nicolay J. Harbin, Katarzyna Kosiek, Morten Lindbæk, Christos Lionis, Carl Llor, Réka Mikó-Pauer, Ruta Radzeviciene Jurgute, Bohumil Seifert, Pär-Daniel Sundvall, Pia Touboul Lundgren, Nikolaos Tsakountakis, Theo J. Verheij, Herman Goossens, Christopher C. Butler, Philippe Beutels, ALIC4Etrial investigators, Emily Bongard, Muirrean de Paor, Slawomir Chlabicz, Maciek Godycki-Cwirko, Nick Francis, Rune Aabenhus, Heiner C. Bucher, Annelies Colliers, An De Sutter, Ana Garcia-Sangenis, Dominik Glinz, Nicolay J. Harbin, Katarzyna Kosiek, Morten Lindbæk, Christos Lionis, Carl Llor, Réka Mikó-Pauer, Ruta Radzeviciene Jurgute, Bohumil Seifert, Pär-Daniel Sundvall, Pia Touboul Lundgren, Nikolaos Tsakountakis

**Affiliations:** 1 Centre for Health Economics Research and Modelling Infectious Diseases (CHERMID), Vaccine and Infectious Disease Institute (VAXINFECTIO), University of Antwerp, Campus Drie Eiken, room D.S.221, Universiteitsplein 1, 2610 Antwerp, Belgium; 2 Julius Center for Health Sciences and Primary Care, University Medical Center, Utrecht, The Netherlands; 3 Interuniversity Institute for Biostatistics and Statistical Bioinformatics (I-BIOSTAT), Data Science Institute (DSI), Hasselt University, Hasselt, Belgium; 4 Laboratory of Medical Microbiology, Vaccine and Infectious Disease Institute (VAXINFECTIO), University of Antwerp, Antwerp, Belgium; 5 Department of Family Medicine and Population Health (FAMPOP), University of Antwerp, Antwerp, Belgium; 6 The Nuffield Department of Primary Care Health Sciences, University of Oxford, Oxford, UK; 7 RCSI Department of General Practice, 123 St Stephens Green, Dublin 2, Ireland; 8 Department of Family Medicine, Medical University of Bialystok, Białystok, Poland; 9 Centre for Family and Community Medicine, Medical University of Lodz, Lodz, Poland; 10 School of Primary Care, Population Sciences and Medical Education, Faculty of Medicine, University of Southampton, Southampton, UK; 11 Section and Research Unit of General Practice, Department of Public Health, University of Copenhagen, Copenhagen, Denmark; 12 Division of Infectious Diseases and Hospital Hygiene, Basel Institute for Clinical Epidemiology and Biostatistics, University Hospital Basel, Basel, Switzerland; 13 Department of Public Health and Primary Care (Centre for Family Medicine), Gent University, Gent, Belgium; 14 University Institute in Primary Care Research Jordi Gol, Via Roma Health Centre, Barcelona, Spain; 15 Department of Clinical Research, Basel Institute for Clinical Epidemiology and Biostatistics, University Hospital Basel, Basel, Switzerland; 16 Department of General Practice, Antibiotic Center for Primary Care, Institute of Health and Society, University of Oslo, Oslo, Norway; 17 Family Doctors’ Clinic, Lodz, Poland; 18 Research Leader Antibiotic Centre for Primary Care, Department of General Practice, University of Oslo, Oslo, Norway; 19 General Practice and Primary Health Care at the School of Medicine, University of Crete, Crete, Greece; 20 Research Unit for General Practice, Department of Public Health, University of Copenhagen, Copenhagen, Denmark; 21 DRC Drug Research Center LLC, Balatonfüred, Hungary; 22 FDC Mano šeimos gydytojas, Klaipeda, Lithuania; 23 Institute of General Practice, First Faculty of Medicine, Charles University in Prague, Prague, Czech Republic; 24 General Practice/Family Medicine, School of Public Health and Community Medicine, Institute of Medicine, Sahlgrenska Academy, University of Gothenburg, Gothenburg, Sweden; 25 Research, Education, Development and Innovation, Primary Health Care, Region Västra Götaland, Sandared, Sweden; 26 Département de Santé Publique, Hôpital de l’Archet, Nice, France; 27 Malia Surgery, Kastelli HC, Heraklio, Greece

**Keywords:** Tamiflu, Cost-utility analysis, Europe, Multi-country, QALY, ILI, Direct cost, Indirect cost, Productivity losses, I19

## Abstract

**Background:**

Oseltamivir is usually not often prescribed (or reimbursed) for non-high-risk patients consulting for influenza-like-illness (ILI) in primary care in Europe. We aimed to evaluate the cost-effectiveness of adding oseltamivir to usual primary care in adults/adolescents (13 years +) and children with ILI during seasonal influenza epidemics, using data collected in an open-label, multi-season, randomised controlled trial of oseltamivir in 15 European countries.

**Methods:**

Direct and indirect cost estimates were based on patient reported resource use and official country-specific unit costs. Health-Related Quality of Life was assessed by EQ-5D questionnaires. Costs and quality adjusted life-years (QALY) were bootstrapped (*N* = 10,000) to estimate incremental cost-effectiveness ratios (ICER), from both the healthcare payers’ and the societal perspectives, with uncertainty expressed through probabilistic sensitivity analysis and expected value for perfect information (EVPI) analysis. Additionally, scenario (self-reported spending), comorbidities subgroup and country-specific analyses were performed.

**Results:**

The healthcare payers’ expected ICERs of oseltamivir were €22,459 per QALY gained in adults/adolescents and €13,001 in children. From the societal perspective, oseltamivir was cost-saving in adults/adolescents, but the ICER is €8,344 in children. Large uncertainties were observed in subgroups with comorbidities, especially for children. The expected ICERs and extent of decision uncertainty varied between countries (EVPI ranged €1–€35 per patient).

**Conclusion:**

Adding oseltamivir to primary usual care in Europe is likely to be cost-effective for treating adults/adolescents and children with ILI from the healthcare payers’ perspective (if willingness-to-pay per QALY gained > €22,459) and cost-saving in adults/adolescents from a societal perspective.

**Supplementary Information:**

The online version contains supplementary material available at 10.1007/s10198-022-01521-2.

## Introduction

Influenza-like-illness (ILI) is defined by the World Health Organization (WHO) as an acute respiratory infection with measured fever of ≥ 38 °C, cough and symptoms onset within the last 10 days [1]. In Europe, ILI usually peaks in winter along with many other viral infections in both adults and children, resulting in large disruptions in people’s daily-life and substantial pressures on both primary and secondary healthcare [2, 3].

Oseltamivir is a neuraminidase inhibitor to treat influenza, which is routinely used for adults and children with ILI in the United States [4]. In Europe, oseltamivir is not often prescribed for patients consulting for ILI in primary care, partly because of the clinical recommendations to only treat “at-risk” groups (e.g. patients with underlying conditions), within 48 h of symptom onset [5]. Moreover, oseltamivir is not reimbursed in many European countries [6], which is likely caused by the debatable clinical evidence and lack of real-world effectiveness [7–11]. Although oseltamivir was approved by the European Medicines Agency in 2002, the effects of oseltamivir on patients’ resource use, productivity losses and health-related quality of life (HRQoL) are still largely unknown [12].

To address the data gaps and collect real-world evidence, a multi-country, multi-season, randomized controlled trial ALIC^4^E (Antivirals for influenza-Like Illness? A randomised Controlled trial of Clinical and Cost-effectiveness in primary CarE, registry number ISRCTN 27908921), was conducted in 15 European countries. This independent, open-label trial aimed to assess the clinical and cost-effectiveness of adding oseltamivir to usual primary care for ILI patients [13, 14]. The primary clinical endpoint analyses demonstrated that the time-to-recovery was 1.02 days [95% Bayesian credible interval 0.74–1.31] shorter in patients treated with oseltamivir [14].

Cost-effectiveness and cost-utility analysis have been commonly used to facilitate decision-making, especially in the drug reimbursement trajectory [15]. Although economic evaluations have been performed to assess the cost-effectiveness of oseltamivir, these studies have been limited primarily to model-based analyses using various secondary aggregated data from literature and focussed on a single country [16–18]. Our study aims to evaluate the cost-effectiveness of adding oseltamivir to usual primary care for adults and children with ILI in 15 European countries, using patient-level cost and HRQoL data collected from the ALIC^4^E trial [14].

## Methods

### A brief summary of the trial

The ALIC^4^E trial was conducted through three consecutive influenza seasons (Q4/2015-Q2/2018) in 15 European countries (details in protocol and primary analysis) [13, 14]. Patients were recruited through 21 primary care networks (including 209 primary care practices) and eligible if they were ≥ 1-year-old, their ILI symptoms onset ≤ 72 h, and would consent to take the antiviral treatment if assigned.

Patients were assigned randomly to usual primary care (hereafter: usual care), or to usual primary care plus oseltamivir (hereafter: oseltamivir) in a 1:1 ratio, stratified by age, severity, time since symptoms onset and comorbidity [13, 14]. Oseltamivir was given twice a day for 5 days with a dosage of 75 mg for adults/adolescents ≥ 13 years, and weight-adjusted dosages for children (≥ 1 year and < 13 years).

A diary was given to each participant to record their symptoms, medication use and self-assessed health using a visual analogue scale (VAS) on a daily basis for 14 days. For children, the diary was filled in by their caregivers. On day 1, 7 and 14, the diary included questions on healthcare visits, impact on patients’ usual daily activities, expenditures, and the EQ5D questionnaire (EQ-5D-5L for adults/adolescents, EQ-5D-Y for children). On day 14 and 28, telephone calls were made to patients to collect data on hospital attendances and to encourage them to complete and return their diaries. On day 28, data on EuroQoL-EQ5D and VAS were also collected during the calls. Since different EQ5D questionnaires were used to estimated health utility, our analyses were conducted separately for adults/adolescents and children.

ALIC^4^E was a large-scale prospective, multi-country and multi-centre, randomised controlled, pragmatic clinical trial, which was designed to investigate the effectiveness and cost-effectiveness of oseltamivir in a real-world setting [13, 14]. It had few restrictions on patients’ recruitment and follow-up after randomisation. Consequently, its generalizability allows the cost and health outcome data collected during the trial to be appropriate for an economic evaluation alongside a clinical trial [19].

### Costs and health-related quality of life

In total, 2212 adults/adolescents and 383 children were included in the cost and HRQoL analyses (Supplement Fig. 1). Average direct and indirect costs per patient for the oseltamivir and usual care arms were estimated in Li et al. [6]. Resource use data was extracted from the patients’ diaries, including: medication use, healthcare visits, hospital attendances (including both day visits and admissions) and hours of productivity losses. The direct cost was estimated by multiplying resource use with itemised country-specific unit costs, which were collected from official tariffs, pharmacies or literature. The human capital approach was used to estimate cost of productivity loss based on the paid-work hour losses. The country-specific list price of oseltamivir is presented in Table 1, and a descriptive analysis including the cost of oseltamivir is available in Supplement Table 2. In summary, the descriptive cost analysis showed that patients treated with oseltamivir had fewer healthcare visits, medication uses, hospital attendances and paid-work hours lost than patients treated without oseltamivir. When including the oseltamivir cost, the average direct costs were higher in adults/adolescents treated with oseltamivir than those without from the healthcare payers’ perspective: €82 [5–100% Credible Intervals (Crl): 72–129] vs €69 [0–95% Crl: 44–84], but the average total costs were lower from a societal perspective (€473 [5–100% Crl: 447–534] vs €476 [0–95% Crl: 405–505]). Amongst children, both average direct costs and total costs were higher in patients treated with oseltamivir (healthcare payers: €73 [5–100% Crl: 63–107] vs. €64 [0–95% Crl: 34–94], and society: €313 [5–100% Crl: 254–479] vs €306 [0–95% Crl: 173–373]). However, these differences were not statistically significant based on one-sided CrI obtained by bootstrapping. In scenario analysis, self-reported costs were used to estimate the average direct costs, where we included costs of healthcare visits, hospital attendance and self-reported amount of spending (e.g., medication). We also included self-reported income losses, where productivity losses were not fully captured (refer as a partial societal perspective).

**Table 1:** List price of oseltamivir: in local currency and euro purchasing power parity(€ PPP) in 2018 value

Country	Currency	Conversion rate to PPP	30 mg (10–15 kg)		45 mg (15–25 kg)		60 mg (25–40 kg)*		75 mg (> 40 kg)	
			Local currency	€ PPP	Local currency	€ PPP	Local currency	€ PPP	Local currency	€ PPP
Poland	PLN	2.56	40	16	70	27	80	31	80	31
Belgium	EUR	1.13	16	14	29	26	32	28	29	26
Spain	EUR	0.93	17	19	22	24	35	37	32	34
UK	GBP	1.01	7	7	15	15	14	14	15	15
Lithuania	EUR	0.66	10	15	17	25	20	30	16	25
Hungary	HUF	201.95	3303	16	6486	32	6607	33	6368	32
Greece	EUR	0.84	11	13	18	21	22	26	18	21
Czech Republic^	CZK	18.15							531	29
Sweden	SEK	12.83	202	16	209	16	404	31	209	16
Denmark^	DKK	10.01	129	13	240	24	258	26	370	37
Netherlands	EUR	1.15	12	10	16	14	24	21	24	21
Norway	NOK	14.59	136	9	218	15	272	19	224	15
Ireland	EUR	1.15	11	9	18	16	22	19	20	18
France	EUR	1.11	12	11	18	16	24	22	24	21
Switzerland	CHF	1.71	30	18	54	32	60	35	86	50

*If the price of 60 mg dosage was not available, the price of 30 mg dosage was multiplied by 2. ^ In Czech Republic and Denmark, no children were recruited in the trial

Incremental quality-adjusted life-years (QALYs) of oseltamivir compared to usual care for adults/adolescents and children from the ALIC^4^E trial are presented in Bruyndonckx et al., by applying a one-inflated beta regression model to estimate QALY gains of oseltamivir use over time [20]. Using the United Kingdom (UK) value set, incremental QALY gains of 0.0006 [95% CrI: 0.0002–0.0010] in adults/adolescents and 0.0007 [95% CrI: -0.0006–0.0021] in children were reported, but the QALY gain in children was not statistically significant [20].

To estimate the incremental cost of oseltamivir compared to usual care, patient-level direct and total (direct plus cost of productivity losses) costs by age were sampled by bootstrapping in each arm (*N* = 10,000 samples/iterations). Similarly, incremental QALYs were bootstrapped from Bruyndonckx et al.’s regression model [20].

### Cost-effectiveness analysis

#### Economic framework

This cost-effectiveness analysis employed both healthcare payers’ (direct costs only) and societal (direct costs and productivity loss) perspectives. Supplement Fig. 2 illustrates the patients’ health care use observed in the trial [14]. The time frame is 14-day from the day patients started oseltamivir (trial’s initial visit) to the last day the diary could be filled in, thus discounting was not applicable. All country-specific costs were expressed in 2018 euro values. If data were extracted from the literature (e.g. hospitalisation costs), costs were inflated to their 2018 values using consumer price indices and then converted to euro using purchasing power parities (PPP) [21, 22]. The ALIC^4^E pragmatic trial provided individual patient data on both costs and effects for two randomised treatment options for an acute illness. Therefore, for this analysis, it was not necessary to develop a decision analytical model to project costs and effects for the options under consideration [19]. The economic analysis plan was included in the study protocol [13] and the analyses were carried out using R version 3.5.0 [13].

#### Outcomes and uncertainty

The expected incremental cost-effectiveness ratio (ICER) of oseltamivir compared to usual care was calculated as the average incremental cost divided by the average QALY gain and was obtained separately for adults/adolescents and children. Apart from the UK, most European countries do not have an explicit official threshold value expressing their willingness-to-pay (WTP) for a QALY gain. We, therefore, show cost-effectiveness acceptability curves and expected value of perfect information for a range of WTP values from €0 to €100,000 per QALY gained (PPP). Uncertainty around average costs and incremental QALY gain was accounted for with probabilistic sensitivity analysis. This allows to express the probability that oseltamivir is (not) cost-effective. Value of information analyses were also carried out by estimating the expected value of perfect information (EVPI). EVPI measures decision uncertainty and accounts for both the probability that an intervention is not cost-effective (probability of making a wrong decision), also the consequences (net loss due to making the wrong decision). EVPI can also be interpreted as the maximum justifiable price for additional studies estimating incremental costs and effects of oseltamivir versus usual care more precisely, to determine its cost-effectiveness with more certainty [23, 24].

#### Scenario analysis

In scenario analysis, self-reported spending was used as an alternative to estimate costs [6]. We also evaluated how the expected ICER would change with a 50% decrease to 20% increase of the country-specific list price of oseltamivir (in € PPP) [25]. Subgroup analyses were carried out to evaluate the cost-effectiveness of oseltamivir among patients with and without comorbidity. Furthermore, country-specific analyses were conducted for adults/adolescents in six countries where the sample size of each arm is > 90 patients: Belgium (*n* = 323), Hungary (*n* = 197), Lithuania (*n* = 188), Poland (*n* = 392), Spain (*n* = 396) and the UK (*n* = 239).

Table 2 illustrates the mean, median and 95% CrI of the incremental cost and QALY for base case, scenario and subgroup analyses. Sample size per age group and per subgroup by treatment arm is available in Supplement Table 1. The subgroup descriptive analysis, and the incremental costs and QALYs per country can be found in Supplement Tables 3 and 4, respectively. Moreover, the primary endpoint of the ALIC^4^E trial, time-to-recovery (in days), was used as health outcome measure in addition to QALY.

**Table 2:** Incremental costs and QALYs of adding oseltamivir to usual care compared to usual care alone, per patient (bootstrap mean *N* = 10,000)

	Perspective	Mean	Median	2.5% credible interval	97.5% credible interval
Adults/adolescents					
Incremental costs **(base case)**	Healthcare payers	12.82	12.90	− 8.38	33.51
	Societal	− 2.68	− 2.47	− 48.89	43.06
Incremental costs (scenario analysis)	Healthcare payers	12.01	12.06	− 9.39	32.79
	Societal	9.40	9.44	− 11.97	30.06
Incremental QALY gained		5.71 × 10^4^	5.71 × 10^4^	2.02 × 10^4^	9.40 × 10^4^
Children					
Incremental costs **(base case)**	Healthcare payers	9.63	11.56	− 30.03	38.28
	Societal	6.18	6.06	− 98.57	112.96
Incremental costs (scenario analysis)	Healthcare payers	6.93	8.82	− 33.38	36.18
	Societal	3.92	5.92	− 38.29	35.32
Incremental QALY gained		7.40 × 10^4^	7.38 × 10^4^	− 5.91 × 10^4^	20.50 × 10^4^
*Subgroup analysis: with comorbidity*					
Adults/adolescents					
Incremental costs	Healthcare payers	40.73	38.45	− 33.74	126.05
	Societal	− 9.40	− 10.08	− 149.52	131.24
Incremental QALY		9.14 × 10^4^	9.13 × 10^4^	− 2.53 × 10^4^	20.94 × 10^4^
Children					
Incremental costs	Healthcare payers	− 146.57	− 139.61	− 499.89	55.82
	Societal	− 223.87	− 200.25	− 732.49	167.65
Incremental QALY gained		− 3.00 × 10^4^	− 2.62 × 10^4^	− 48.41 × 10^4^	40.27 × 10^4^
*Subgroup analysis: without comorbidity*					
Adults/adolescents					
Incremental costs	Healthcare payers	7.32	7.61	− 13.55	26.54
	Societal	− 1.26	− 1.07	− 51.97	47.48
Incremental QALY gained		5.01 × 10^4^	5.00 × 10^4^	1.25 × 10^4^	8.76 × 10^4^
Children					
Incremental costs	Healthcare payers	24.94	24.34	8.68	44.11
	Societal	30.52	30.32	− 74.71	139.37
Incremental QALY gained		8.79 × 10^4^	8.79 × 10^4^	− 4.90 × 10^4^	22.52 × 10^4^

Costs in euro purchasing power parity (€PPP) 2018 value

*QALY* quality-adjusted life-year, *EUR (PPP)* purchasing power parity in euro

## Results

ICERs using QALYs gained as health outcomes are presented in this section, whereas ICERs using time-to-recovery (in days) are reported in Supplement Fig. 3.

### Base case analysis

**Fig. 1:**
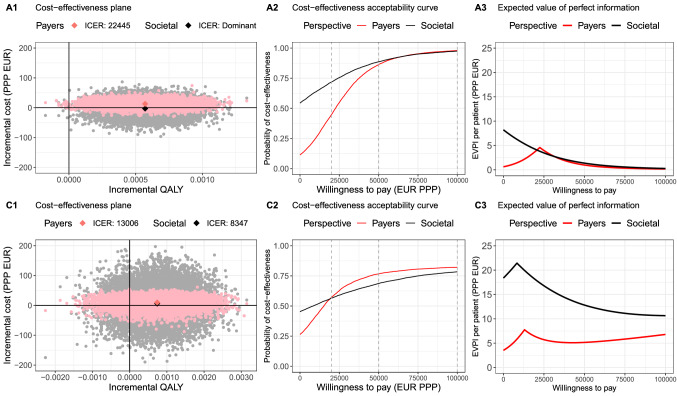
Base case analysis results among (A) adult/adolescent (top row) and (C) child (bottom row) ILI-patients from two perspectives: payers’ (red dots or lines) and societal (black dots or lines) perspectives. The left column: cost-effectiveness plane, middle column: cost-effectiveness acceptability curve, right column: expected value of perfect information. *QALY* quality-adjusted life-year, *EUR (PPP)* purchasing power parity in euro, *Payers* healthcare payers, *EVPI* expected value of perfect information

For adults/adolescents with ILI, the expected ICER of oseltamivir compared to usual care alone is €22,445 per QALY gained from the healthcare payers’ perspective. From a societal perspective, adding oseltamivir to usual care dominates usual care alone (cost-saving and results in QALY gains, Fig. 1A1). For children, the expected ICERs of oseltamivir versus usual care are €13,006 and €8,347 from for the healthcare payers’ and societal perspective, respectively (Fig. 1C1).

The cost-effectiveness planes in Fig. 1 (left plots) show the uncertainties around incremental costs and QALYs, while the cost-effectiveness acceptability curves (CEAC) in Fig. 1 (middle plots) present the probability that oseltamivir is cost-effective for a range of WTP values. For adults/adolescents, there is 11% and 54% chance that oseltamivir is cost-saving versus usual care from healthcare payers’ and societal perspectives, respectively (Fig. 1A1 and A2), but also a 0.14% chance that oseltamivir results in QALY losses from both perspectives (Fig. 1A1). Uncertainty around incremental costs from the societal perspective is larger due to the additional uncertainty around costs of productivity losses (Fig. 1A1). Given WTP values of €20,000, 50,000 and 100,000 per QALY gained, oseltamivir has probabilities of 45%, 86% and 98% to be cost-effective compared to usual care from the healthcare payers’ perspective, and 72%, 89% and 97% from the societal perspective (Fig. 1A2). For children, the cost-effectiveness of oseltamivir versus usual care is more uncertain. Compared to adults/adolescents, oseltamivir has a higher chance to be cost-saving in children (26%) from the healthcare payers’ perspective, yet a lower chance (45%) from the societal perspective (Fig. 1C1 and C2). Children’s use of oseltamivir also has a higher chance to result in QALY losses (14% for both perspectives, Fig. 1C1).

The extent of decision uncertainty is expressed as EVPI over a range of WTP values and presented in Fig. 1 (right plots). In adults/adolescents, EVPI peaks at a WTP value of €22,445 per QALY gained for the healthcare payers’ perspective (Fig. 1A3), because this WTP value leads to the highest decision uncertainty: if WTP > €22,445 per QALY gained, oseltamivir is cost-effective, otherwise, oseltamivir is not cost-effective. At this WTP value, €4.6 per patient is the maximum justifiable price for conducting additional research to measure the incremental costs and QALY’s of oseltamivir more precisely to decide whether oseltamivir is cost-effective or not. From the societal perspective, oseltamivir is cost-effective for all WTP values considered, but decision uncertainty decreases from €8.2 per patient to €0.3 with increasing WTP values (Fig. 1A3). In children, the decision uncertainty is larger from the societal than the healthcare payers’ perspective, which is consistent with the findings shown in the cost-effectiveness plane and CEACs (Fig. 1C). The EVPI peaks at €21.4 and €7.8 at WTP values of €8,347 and €13,006 per QALY from the societal and healthcare payers’ perspectives, respectively.

### Scenario analysis

**Fig. 2:**
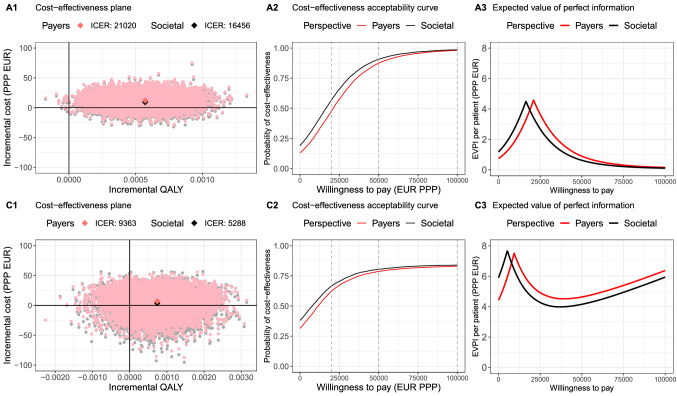
Scenario analysis results in (A) adult/adolescent (top row) and (C) child (bottom row) ILI-patients from two perspectives: both payers’(red dots or lines) and societal (black dots or lines) perspectives. Left column: cost-effectiveness plane, middle column: cost-effectiveness acceptability curve, right column: expected value of perfect information. *QALY* quality-adjusted life-year, *EUR (PPP)* purchasing power parity in euro, *Payers* healthcare payers, *EVPI* expected value of perfect information

When using self-reported spending to estimate costs, the results from the healthcare payers’ and partial societal perspectives were very similar (Fig. 2). Compared with the base case analysis, Fig. 2 demonstrates that the expected ICERs were lower from the healthcare payers’ perspective in both age groups. From the partial societal perspective, oseltamivir is not dominant in adults/adolescents, but has a lower ICER in children versus the base case.

### Price sensitivity analysis

**Fig. 3:**
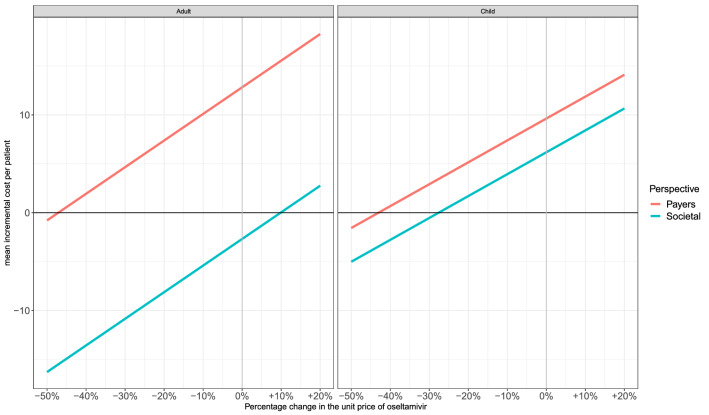
Price sensitivity analysis (base case assumptions for other input parameters). *Payers* healthcare payers

The price sensitivity analysis shows that oseltamivir would become cost-saving at a 50% decrease in price for adults/adolescents from the healthcare payers’ perspective (Fig. 3, left plot). A 10% price rise would no longer render oseltamivir cost-saving from the societal perspective (ICER = €71, Fig. 3, left plot). In children, oseltamivir would become cost-saving at a 50% and 30% price reduction, from the healthcare payers’ and societal perspective, respectively.

### Subgroup analysis: patients with and without comorbidity

**Fig. 4:**
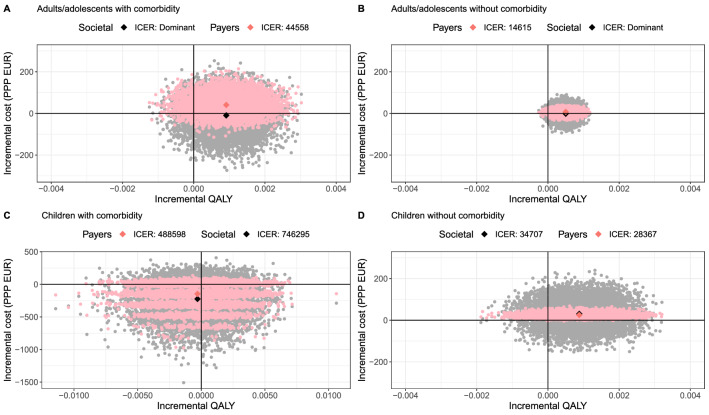
Subgroup analysis of adults/adolescents and children with and without relevant comorbidity. The scales of the *x*-axis and *y*-axis of graph C ‘children with comorbidities’ are different from the other graphs. Relevant comorbidity was: heart disease, diabetes, chronic respiratory condition, hepatic, haematological, neurological, or neurodevelopmental condition, stroke or transient ischaemic attack, or overnight hospital stay in the previous year. *QALY* quality-adjusted life-year, *Payers* healthcare payers

In adults/adolescents with relevant comorbidity, oseltamivir is cost-effective up to WTP values of €44,558 from the healthcare payers’ perspective, and is cost-saving from the societal perspective (Fig. 4A). In children with comorbidity, oseltamivir would save costs from both perspectives, yet, it would also lead to QALY losses (see South West quadrant in Fig. 4C). In adults/adolescents without comorbidity, the expected ICER is €14,615 from the healthcare payers’ perspective, which is lower than the base case ICER (€22,445). Oseltamivir dominates from a societal perspective (see South East quadrant in Fig. 4B). Contrarily, in children without comorbidity, the expected ICER is €28,367 and €34,707 from the healthcare payers’ and societal perspective, respectively, two fold and four fold higher compared with the base case ICERs (Fig. 4D).

For adults/adolescents and children without comorbidity, the extent of decision uncertainty is similar as in base case analysis (Supplement Fig. 4). The EVPI in adults/adolescents peaks at €4 and €9 per patient for the healthcare payers’ and societal perspective, respectively (Supplement Fig. 4B), which is € 9 and €24 per child (Supplement Fig. 4D). In adults/adolescents and children with comorbidity, the uncertainty around oseltamivir being cost-effective is much larger, especially in children (Fig. 4A and C, wide spread of dots). In adults/adolescents, EVPI peaks at €19 and €24 per patient for the health care payers’ and societal perspective, respectively (Supplement Fig. 4A).

Such uncertainties are likely caused by the small sample size of the subgroup (bootstrapping from 45 children with relevant comorbidity). Moreover, one child receiving usual care was hospitalised, which resulted in a few layers in the cost-effectiveness plane (Fig. 4C). Each layer represents a group of mean values based on a bootstrap sample that sampled the hospitalised child a number of times. The more frequent this hospitalised child was sampled for a single bootstrap, the higher the mean of direct cost in the usual care arm became, consequently, the lower the incremental cost.

### Country-specific analyses

**Fig. 5:**
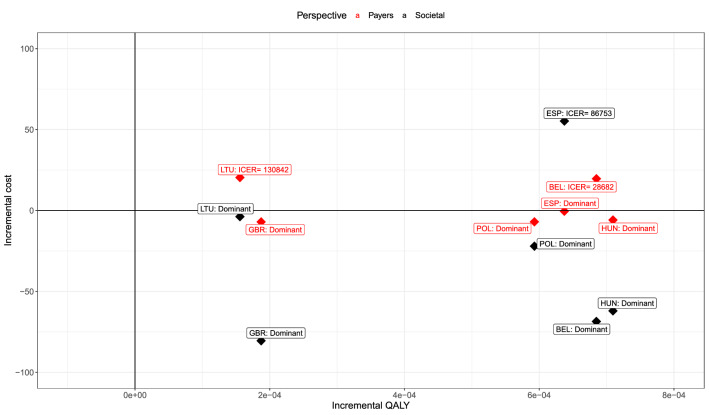
Cost-effectiveness plane: incremental cost-effectiveness ratios from the healthcare payers’ and societal perspectives in adults/adolescents with ILI in six European countries. Red dots and text represent the healthcare payers’ perspective. Black dots and text represent the societal perspective. ISO3 codes: *BEL* Belgium, *ESP* Spain, *GBR* the United Kingdom, *POL* Poland, *HUN* Hungary and *LTU* Lithuania. *QALY* quality-adjusted life-year, *EUR (PPP)* purchasing power parity in euro, *Payers* healthcare payers, *EVPI* expected value of perfect information

For six countries, the expected ICERs are plotted in Fig. 5. Oseltamivir treatment resulted in average incremental QALY gains in all six countries versus usual care alone. In the UK, Poland and Hungary, oseltamivir is cost-saving from both the healthcare payers’ and societal perspectives. In Belgium and Lithuania, oseltamivir is cost-saving from the societal perspective, and the expected ICERs from the healthcare payer’s perspective are €28,682 and €130,842, respectively. In Spain, oseltamivir is cost-saving from the healthcare payers’ perspective, but the expected ICER from the societal perspective is €86,753, due to much higher productivity costs in the oseltamivir arm. The cost-effectiveness planes including uncertainty (Supplement Figs. 6–11) show that oseltamivir has a probability to be cost-saving from both perspectives for all six countries (except for Belgium from the healthcare payers’ perspective), but also a probability to result in QALY losses.

The decision uncertainty per country was measured with EVPI in Supplement Fig. 6–11. For Belgium and the UK, decision uncertainty is larger from the healthcare payers’ perspective than from the societal perspective. For the four other countries, decision uncertainty from the societal perspective is largest. In Belgium, the decision uncertainty is lowest: EVPI ranges between €0 and €4.5 depending on the WTP value and perspective (Supplement Fig. 6C). In Lithuania, the cost-effectiveness of oseltamivir treatment is surrounded with most decision uncertainty, especially from the societal perspective (EVPI between €33–36 per patient, depending on WTP value, Supplement Fig. 11C).

## Discussion

This is the first economic evaluation investigating the cost-effectiveness of adding oseltamivir to primary care for ILI alongside an open-label, randomised controlled trial, including patient-level data of both adults and children from all four European regions (north, west, south and east Europe). Using a consistent method across 15 countries, while accounting for countries’ healthcare systems and healthcare seeking behaviours, oseltamivir showed a reduction in healthcare resource use and an improvement in patients’ quality-adjusted life-years. We estimated the expected ICERs of oseltamivir at €22,445 in adults/adolescents and €13,006 in children. We found oseltamivir to be cost-effective if the WTP exceeds €22,500 per QALY gained for European healthcare payers. From a societal perspective, oseltamivir is cost-saving in adults/adolescents, and cost-effective in children at WTP ≥ €8,400 per QALY gained. When focusing on self-reported costs in the scenario analysis, the differences between the healthcare payers’ and partial societal perspectives were smaller, because the self-reported income losses of patients or caregivers do not fully capture all productivity losses incurred under a full societal perspective.

### Comparison with literature

A modelling study of oseltamivir treatment in different pandemic scenarios among healthy 13–64 year olds in the UK concluded that oseltamivir was cost-effective from the National Health Service (NHS) perspective and cost-saving from a societal perspective [26]. For the UK, we had similar findings in patients ≥ 13 years, although our healthcare payers’ perspective is broader than that of the NHS, by having included patients’ medication costs. Wailoo et al. evaluated three NIs in 12–65 year old ILI patients from the NHS perspective and reported the ICER of oseltamivir (administered ≤ 48 h) at £32,406 compared with no treatment, whereas our study found oseltamivir dominant over a 14-day time horizon [27]. Postma et al. found oseltamivir to be cost-saving compared with usual care in patients with chronic disease in Dutch adults/adolescents, which is consistent with the findings from the societal perspective in our trial [28].

We also compared our study with several model-based analyses outside of Europe. A Brazilian study assessed the cost-effectiveness of oseltamivir for influenza (N1H1) in a high-risk population (i.e. older adults, children and people with chronic diseases or immunodeficiency), and concluded that oseltamivir was cost-saving from the Brazilian healthcare payer’s perspective [29]. In contrast, our healthcare payers’ perspective analysis showed that oseltamivir has higher ICERs per QALY gained among adults/adolescents with comorbidity (€44,558) and older adults aged over 65 years (€352,172), albeit with large uncertainties. Sample sizes of these subgroups were relatively small (*N* = 365 and *N* = 201, respectively), with fewer hospital admissions and no intensive care admission being observed in the ALIC^4^E subgroups. Nakagawa and colleagues performed a cost-effectiveness analysis in the Japanese adult outpatient setting [30]. Similar to our study, it reported that oseltamivir was cost-effective from a payer’s perspective. However, a rapid diagnostic test is frequently performed for influenza-suspected cases in Japan [31]. It is worth noting that our analysis included all ILI patients (including non-influenza patients) in the European primary care setting, where laboratory or point-of-care diagnostics for influenza are rarely performed.

A modelling study in the United States evaluated the cost-effectiveness of oseltamivir in children under 18 years with uncomplicated influenza. It found that standard use of oseltamivir (without diagnostic test) in these patients was cost-effective (WTP: USD 100,000 per QALY) from the healthcare payers’ perspective, but their “test and treat only positive cases with oseltamivir” strategy had a lower, more favourable, ICER than standard use [32]. Along with country-specific issues such as pricing, this might explain the differences between our analysis for ILI patients and the other studies focusing on influenza patients only.

### Strengths and limitations

To our knowledge, only one multi-country analysis exist for England, France and Germany [33] and none alongside a randomised clinical trial, although many single country model-based analyses have been done to estimate the cost-effectiveness of oseltamivir [27, 28, 34, 35]. Differences in healthcare system organisation, resource use and unit costs between countries render multi-country analyses challenging. We have provided overall and country-specific cost-effectiveness estimates (given n > 90 per arm), while adopting both the healthcare payers’ and societal perspectives, yielding a firm basis to help inform recommendations and reimbursement decisions.

Our analysis has several other strengths. First, both cost and health outcomes were directly collected from individual patients, and the comparisons are made from randomised patients. Hence, it reflects an unbiased estimate of treatment effect, and it is suitable for economic evaluation without constructing a model [20]. Since our evaluation is based on real-world data, it requires fewer assumptions, a less diverse range of data sources and it increases internal validity versus a model-based approach. Next, in addition to QALYs, we used the time-to-recovery, the ALIC^4^E trials’ primary endpoint, as an outcome measure showing the ICER per day of faster recovery, which can be insightful for clinicians and patients (Supplement Fig. 3). Furthermore, in the absence of an explicit WTP threshold for most individual European countries or at the European level, we have presented our results over a range of WTP values, to facilitate external value judgements for policy making. We explored conceptual uncertainty from different perspectives and data sources, parametric uncertainty in PSA and decision uncertainty using EVPI, to inform decision makers on the certainty of oseltamivir being cost-effective and the consequence of making a wrong decision [36].

Conservative approaches were used in our analysis which might underestimate the cost-effectiveness of oseltamivir. As patients were recruited via primary care practices, patients with very severe illness were less likely to be recruited, as they might be less willing to be enrolled in the trial. The ALIC^4^E trial did not capture any ILI-associated intensive care admissions or death (sample size > 3000). Next, paid-work hours losses were used to estimate the cost of productivity losses under the human capital approach. The hours lost on other productive activities (e.g. education, volunteering, household) were not included. Moreover, our study used a time horizon of 14-day, which is consistent with the WHO definition of ILI, while the possible long-term consequences of ILI might not have been fully captured. Furthermore, we did not attempt to quantify the indirect effects of using oseltamivir, which might reduce the duration of infectivity, therefore, limit further transmission. Finally, according to literature, hospitalisation of ILI patients with comorbidities often result in higher costs, but as we used country-specific average hospitalisation costs, results in the subgroup analyses specifically focusing on patients with comorbidities might be underestimated [34].

There are also a few limitations. First, it is very challenging to use a non-disease specific, preference-based questionnaire (EQ5D) to evaluate the QALY for acute infectious diseases, especially for very young children. We used the EQ-5D-3L UK value set to estimate the QALY for children in the absence of a specific EQ-5D-Y value set, which might create methodological uncertainties around the results in children. The EQ-5D-5L and EQ-5D-3L UK value sets were used in our analyses to estimate incremental QALY gains. In Bruyndonckx et al., we previously investigated two additional scenario analyses for the ALIC^4^E QALY estimates: (1) using country-specific value sets, when available, and using the average of these country-specific value sets for the other countries, and (2) using the average of the available value sets for all participating countries. Both approaches produced similar results as using only the UK value set [20]. Second, recall bias cannot be avoided as data were collected from participants. Third, the trial was not powered to capture differences in cost-effectiveness between subgroups and countries. Due to the sample size, we were unable to perform country-specific analyses for all 15 countries in adults/adolescent and children. Fourth, by definition a cost-effectiveness analysis alongside a clinical trial uses data from a single trial and does not integrate efficacy measures from diverse sources into a simulation model. It is, therefore, reassuring that the overall findings of the ALIC^4^E trial were in line with those reported on the efficacy of oseltamivir in three meta-analyses [7, 37, 38]. Lastly, our analysis applies to normal seasonal epidemics and a non-pandemic situation. Oseltamivir’s indirect effect and associated value may increase substantially when influenza pandemics should occur. Given its value in non-pandemic years, upscaling of production capacity might be considered in the event of a future influenza pandemic.

## Conclusion and implications

In influenza season, adding oseltamivir to usual primary care is likely to be cost-effective for treating patients of all ages with ILI from the healthcare payers’ perspective, if the WTP value is above €22,445. From a societal perspective, oseltamivir is cost-saving in adults/adolescents and cost-effective in children (WTP > €8,347). Uncertainties in the cost-effectiveness of oseltamivir for subgroups of patients, especially children, with comorbidities, were observed. Specific analyses showed that oseltamivir would be cost-saving from healthcare payers’ perspective in four out of six countries investigated.

Our analysis demonstrated that oseltamivir is a cost-effective treatment up to 72 h after symptom onset in all ILI patients presenting to primary care during influenza seasons in European countries, regardless of influenza diagnostic test results. Decision makers at European and country-level might consider the reimbursement of oseltamivir. Clinicians might consider adding oseltamivir to primary care for ILI given the combination of acceleration in recovery-time, improvement in quality of life and the associated economic benefits for individual patients, healthcare system, as well as the society.

## Supplementary Information

Below is the link to the electronic supplementary material.

Supplementary file1 (PDF 116 KB)

## Data Availability

Almost all the data analysed and generated during this study are included in this published article and its supplementary material files. Formal requests for additional data can be made to the co-responding author (XL) or the senior author (CCB, PB) using a bespoke data request form delineating research aims, methods, and the variables.

## Abbreviations

ILI: Influenza-like-illness
WHO: World Health Organization
HRQoL: Health-related quality of life
ALIC^4^E: Antivirals for influenza-Like Illness? A randomised Controlled trial of Clinical and Cost-effectiveness in primary CarE, registry number ISRCTN 2790892
QALY: Quality-adjusted life-year
CrI: Credible interval
VAS: Visual analogue scale
PPP: Purchasing power parities
ICER: Incremental cost-effectiveness ratio
EVPI: Expected value of perfect information
CEAC: Cost-effectiveness acceptability curves
WTP: Willingness-to-pay
UK: United Kingdom

## Code availability

Formal requests for R codes can be made to the co-responding author (XL) or the senior author (CCB, PB) using a bespoke data request form.
